# Improving bactericidal performance of implant composite coatings by synergism between Melittin and tetracycline

**DOI:** 10.1007/s10856-022-06666-3

**Published:** 2022-05-21

**Authors:** Vahid Zarghami, Mohammad Ghorbani, Kamran Pooshang Bagheri, Mohammad Ali Shokrgozar

**Affiliations:** 1 Institute for Nanoscience & Nanotechnology, Sharif University of Technology, Tehran, Iran; 2 Department of Materials Science and Engineering, Sharif University of Technology, Tehran, Iran; 3 Venom & Biotherapeutics Molecules Lab., Medical Biotechnology Department, Biotechnology Research Center, Pasteur Institute of Iran, Tehran, Iran; 4 National Cell Bank of Iran, Pasteur Institute of Iran, Tehran, Iran

## Abstract

Methicillin resistance *Staphylococcus aureus* bacteria (MRSA) are serious hazards of bone implants. The present study was aimed to use the potential synergistic effects of Melittin and tetracycline to prevent MRSA associated bone implant infection. Chitosan/bioactive glass nanoparticles/tetracycline composite coatings were deposited on hydrothermally etched titanium substrate. Melittin was then coated on composite coatings by drop casting method. The surfaces were analyzed by FTIR, XRD, and SEM instruments. Tetracycline in coatings revealed multifunctional behaviors include bone regeneration and antibacterial activity. Releasing ALP enzyme from MC3T3 cells increased by tetracycline, so it is suitable candidate as osteoinductive and antibacterial agent in orthopedic implants coatings. Melittin increased the proliferation of MC3T3 cells. Composite coatings with combination of tetracycline and Melittin eradicate all MRSA bacteria, while coatings with one of them could no t eradicate all of the bacteria. In conclusion, chitosan/bioactive glass/tetracycline/Melittin coating can be suggested as a multifunctional bone implant coating because of its osteogenic and promising antibacterial activity.

**Figure:**
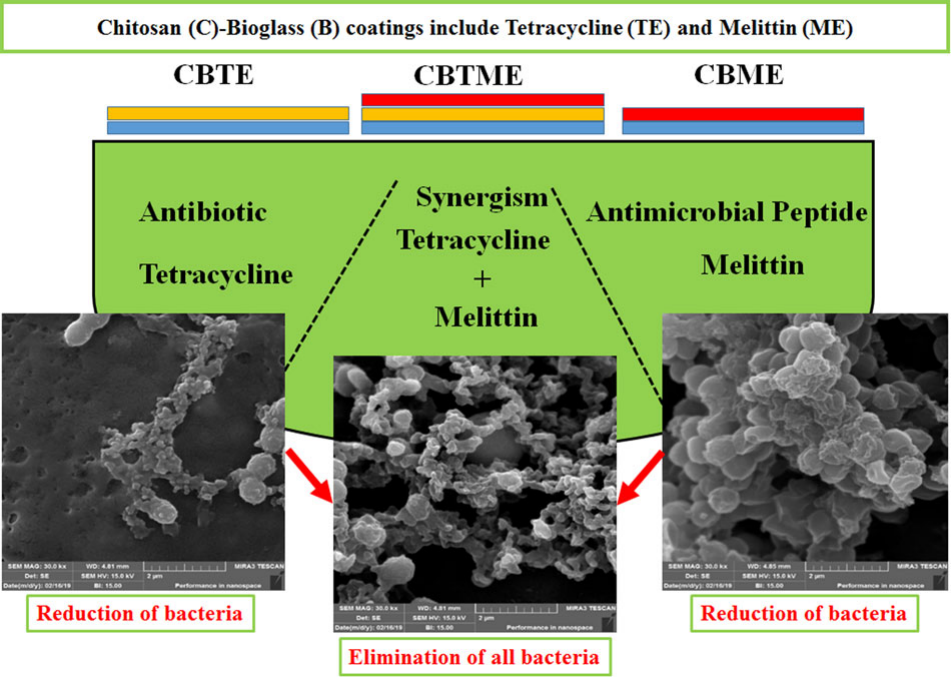
Graphical abstract

Graphical abstract

## Introduction

Recently, synthesis of multifunctional biomaterials have received considerable attention due to their significant potential applications [1, 2]. In this regard, many studies have been focused on designing of multifunctional nanostructures, coatings, scaffolds [3–6]. There are various combinations of bioactive and bio-inert components to improve or control the interaction of materials with their surrounding biological environment [7, 8]. In the field of orthopedics, multifunctional coatings or scaffolds are widely developed for simultaneously promoting osseointegration and antibacterial properties [9–11]. Aseptic loosening, infection and bacterial biofilms are in particular problems of bone implants [11–13]. To eradication of bacterial infection and prevent biofilm formation on the implants, antibacterial metal ions such as Ag and Cu, and antibiotics such as vancomycin and gentamycin are widely used [14–17]. Release of antibacterial metal ions caused poor proliferation and differentiation of host cells and loosening osseointegration [18]. Also long-term use of conventional antibiotics always causes bacterial resistance [19]. In this critical condition, development, design, or discovery of antimicrobial peptides (AMPs) have received much attention to dealing with antibiotic resistant bacteria and biofilm associated infections [20–27].

Melittin, an antimicrobial peptide of 26 amino acid residues, is the main component of honey bee venom with potent antibacterial activity [28–30]. Melittin induces pores in membranes and has a suitable synergistic effect with antibiotics on killing the resistant bacteria and, also, inhibiting biofilm formation [23, 28, 31–34].

Tetracycline a polyketide class and primarily bacteriostatic antibiotic is commonly used against a wide range of Gram-positive bacteria. Also, tetracycline is effective in the modulation of the immunoinflammatory imbalance and enhancing bone formation, decreasing connective tissue breakdown and diminishing bone resorption [35, 36]. But tetracycline is not effective against MRSA and cannot eradicate MRSA bacteria in bone implant infections [37].

Chitosan/ bioactive glass composite coatings have combined the flexibility of chitosan with strength and bioactivity of bioactive glass particles. Therefore these composites have emerged recently as new class of implant coatings with interesting properties include improved mechanical, physiochemical and biological properties [38].

Chitosan has intrinsic antibacterial activity against *Staphylococcus aureus* gram positive bacteria and immunomodulatory effect for accelerating angiogenesis [39, 40]. In comparison to synthetic polymers, chitosan is similar to the chemical composition of the extracellular matrix of biological systems and it can be degraded by enzymatic degradation [41]. Also, chitosan has the ability to form close-fitting films on the surface of implantable metals, ceramics and polymers which is not usually possible with other biopolymers [42]. Bioactive glass ceramics with osteogenic and bonding properties to living bone are commercially used as bone graft. Also bioactive glass matrices have been used as dental implants, bone fixation devices, and implant coatings [43]. The surface features (wettability, bioactivity, cell adhesion, and osteoconductive functioning) of the bioactive glass have been enhanced by reduction of particle size. Consequently, nanometer grain size and surface wettability of nanosized bioactive glass have led to improved selective vitronectin adsorption (a protein that mediates osteoblast adhesion), followed by modulation the promoted osteoblast adhesion and long-run functioning [43–45].

In the present study, we used bioactive glass nanoparticles as osteogenic agent and chitosan polymers as reservoir. According to the abovementioned issues, this study was aimed to investigate the applicability of synergism between Melittin and tetracycline as antibacterial agents in eradication of bone associated methicillin resistance *Staphylococcus aurous* bacteria (MRSA) infections and bone cell formation ability of coatings. Accordingly, the physiochemical, cell proliferation and differentiation and antibacterial properties of coatings were investigated.

## Materials and methods

### Coating procedure

#### Etching of titanium

Biomedical grade titanium foils (Alfa Aesar, USA) with dimensions of 5 mm × 5 mm × 0.7 mm were hydrothermally etched in H_2_O_2_ aqueous solution (6% v/v) in 170 °C for 6 h. Teflon-lined stainless steel autoclave was used for etching process and was filled up to 70%. The Ti specimens were taken out after cooling down to room temperature, ultrasonically washed by acetone, ethanol and pure water respectively and allowed to dry in ambient air.

#### Chitosan-based composite coating

Low molecular weight chitosan powders with 75–85% degree of deacetylation (Mw = 50,000–190,000 Da; Sigma-Aldrich: Germany) were dissolved at 6 g/l in 0.25% acetic acid aqueous solution. Wet synthesized bioactive glass nanoparticles with a nominal composition of (wt.%) 45 SiO_2_, 49 CaO, 6 P_2_O_5_ with a medium particle size of 100 nm (BGNs; Nik Ceram Razi biomedical engineering, Iran) and tetracycline (Tet; Jaber Ebn Hayyan, Iran) powders were added into the CS solution to prepare composite suspensions at proportional concentrations of antibiotic and nanoparticles. Magnetic stirring was applied for 24 h for dispersion and homogenization was done by sonication for 30 min immediately before coating. 30 µl of the solution was drop-casted over the titanium samples and then dried at ambient conditions for 24 h.

#### Peptide synthesis and coating

Melittin (GIGAVLKVLTTGLPALISWIKRKRQQ) was synthesized at DgPeptides Company (Hangzhou, China) using Fmoc chemistry. The peptide was ordered to be amidated from its C-terminal at the purity of ≥95% confirmed using Reverse-Phase HPLC by the company. Mass spectrometry was performed by the company to confirm the molecular weight of synthetic Melittin. The peptide concentration and its purity was respectively reconfirmed using bicinchoninic acid assay (BCA) and RP-HPLC as described earlier [46]. Four µl of Melittin was drop-casted over coatings and dried at ambient conditions for 3 h.

#### Experimental groups

Experimental groups and the composition of them are shown in Table 1.

**Table 1:** The composition of experimental groups

Row	Group name	Composition			
		Chitosan (µg/µl)	Bio-glass (µg/µl)	Tetracycline (µg/µl)	Melittin (µg/µl)
1	Ti	–	–	–	–
2	CS	6	–	–	–
3	CBG	6	6	–	–
4	CBTE	6	6	1.28	–
5	CBME	6	6	–	0.075
6	CBTME	6	6	0.16	0.025

*Ti* titanium, *CS* chitosan, *CBG* chitosan/bioactive glass, *CBTE* chitosan/bioactive glass/tetracycline, *CBME* chitosan/bioactive glass/melittin, *CBTME* chitosan/bioactive glass/tetracycline/melittin

### Surface characterization

Scanning electron microscopy (JSM-7610F, JEOL Co., Japan and MIRA3, TESCAN Co., Czech) was used for observation of coatings’ morphology and microstructure. Surface roughness before and after hydrothermal etching was measured using a stylus instrument (MAHR POCKET SURF EMD-1500-311). The crystal structure of hydrothermally etched surface was analyzed by grazing method of X-ray diffraction diffractometer (XRD, Philips, PW1730, Cu-Kα). Fourier transform infrared spectroscopy (FTIR, ABB BomemMB100, USA) in the range of 400–4000 cm^−1^ with a resolution of 4 cm^−1^ was utilized to identify the interaction between the coatings component.

### Cell culture assays

#### Cell culture

Preosteoblast MC3T3-E1 cell line was obtained from the national cell bank (Pasteur Institute of Iran, Tehran, Iran) and cultured in α-MEM supplemented with 10% fetal bovine serum, 100 IU/ml penicillin, and 100 µg/ml streptomycin at 37 ^o^C in a humidified atmosphere with 5% CO_2_ Proliferation medium supplemented with 1% L-ascorbic acid-2-phosphate 20 mM, 1% β- glycerophosphate 1 M and 0.00004% dexamethasone (1 mg/ml) was used for investigation of differentiation. Once confluent, the cells were collected by trypsinization of the adherent cells and resuspended in the medium. The cells were then counted using the trypan blue dye.

#### Cell proliferation assay

Cell proliferation was evaluated using the standard Alamar Blue assay protocol. Briefly, 10,000 cells were seeded on the specimens and incubated at 37 ^o^C in 5% CO2 for 1, 3, and 7 days. After each interval, the medium discarded and new medium containing 10% of 440 mM sterile solution of resazurin in PBS was added in each well and the cells were incubated for 4 h. Absorbance was then measured at 570 nm and also 630 nm as a reference wavelength by an ELISA reader (BioTek microplate reader, USA).

#### Live/dead assay

After 2 days of cell culture, the media were discarded and specimens were washed with PBS twice. Then 200 µl of fresh medium containing 4 μM calcein AM and 10 μM ethidium homodimer-1 (Life Technologies, UK) was added to each well and incubated at 37 ^o^C for 30 min. A fluorescence microscope (Olympus BX51, Japan) was employed for live/dead assay. Live and dead cells were respectively observed in green and red colors.

#### Alkaline phosphatase activity and DNA content assays

MC3T3 Cells were seeded onto specimens in differentiation medium. Every 2 days, the media above specimens were replaced by fresh media. Alkaline phosphatase (ALP) activity was determined on days 3, 7 and 14 after seeding of cells onto specimens. In each time point, the cells were treated by 0.5% Triton X-100 in PBS, were frozen at −80 °C, and then were thawed. Freeze-thawing was repeated three times. The solutions above specimens were assayed for ALP activity using an ALP assay kit (pNPP: Sigma-Aldrich). Enzyme activity was normalized against the total DNA content determined by Picogreen kit (Invitrogen) according to the manufacturer’s instruction.

### Antibacterial assay

#### Determination of antimicrobial potential of the experimental groups

Antimicrobial activity of the specimens was tested against Methicillin-resistant *Staphylococcus aureus* (MRSA) bacteria strain according to our previous study [46]. Briefly, MRSA bacteria were cultured overnight. Fifty µl of bacteria solution was transferred into sterile tubes containing 2 ml of Mueller Hinton Broth (MHB) and incubated at 37 ^o^C for 2 h to obtain bacteria in the mid-logarithmic phase of growth. Bacterial suspensions were prepared by spectrophotometry at 625 nm. According to the 0.5 McFarland standard, the optical density in the range of 0.08–0.1 is equivalent to 1.5 × 10^8^ CFU/ml. To increase the accuracy of quantification, OD of the suspension was considered at 0.09 [47]. The numbers of bacteria was adjusted to 1.5 × 10^4^ CFU/ml by diluting in the same medium prior to use and seeded on the specimens (three specimens per group for each time point) and incubated at 37 °C. After 6 h, the residual planktonic bacteria were plated on Mueller Hinton Agar (MHA) and incubated at 37 °C overnight and the resultant colonies were counted. For adherent bacteria, the bacterial suspension above the specimens was discarded and replaced with 200 µl PBS. The specimens were sonicated for 30 s in PBS to detach the adherent bacteria from the surfaces. The suspension was cultured on MHA as mentioned above.

#### Morphological evaluation by field emission-scanning electron microscopy

To visualize changes in cell morphology due to the effects of specimens on MRSA strain, scanning electron microscopy (SEM) was performed. The Bacteria were fixed by 2.5% glutaraldehyde in PBS and then dehydrated by 10, 30, 50, 70, 90, 100% ethanol solutions sequentially. The samples were allowed to dry at room temperature, coated with gold nanoparticles through an automatic sputter coater, and visualized using FE-SEM instruments (JSM-7610F, JEOL Co., Japan and MIRA3, TESCAN Co., Czech).

### Statistical analysis

The data are presented as mean ± standard deviation (SD). The results were analyzed using ANOVA with Tukey’s post hoc *t*-test.

## Results

### Characterization of surfaces

**Fig. 1:**
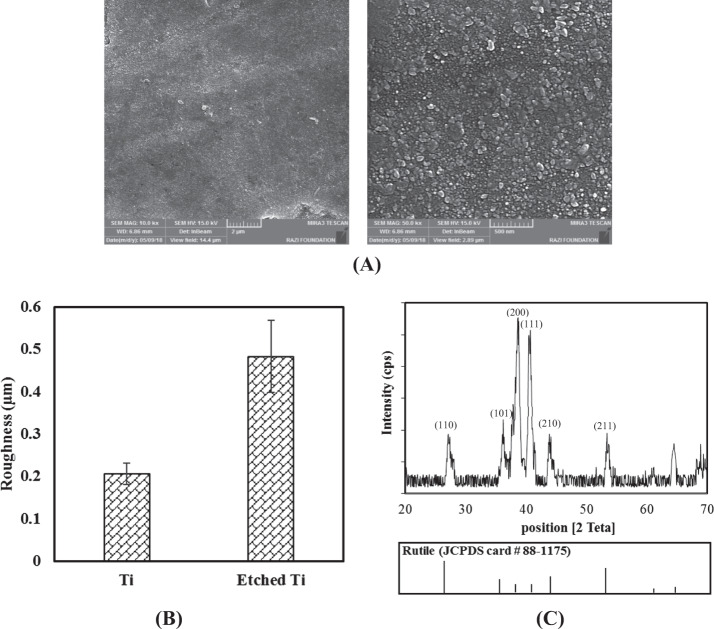
**A** FE-SEM images of biomedical grade Titanium sheets after hydrothermal etching at 10 kx and 50 kx magnification. **B** Surface roughness changes. **C** GIXRD pattern of hydrothermally etched Ti

**Fig. 2:**
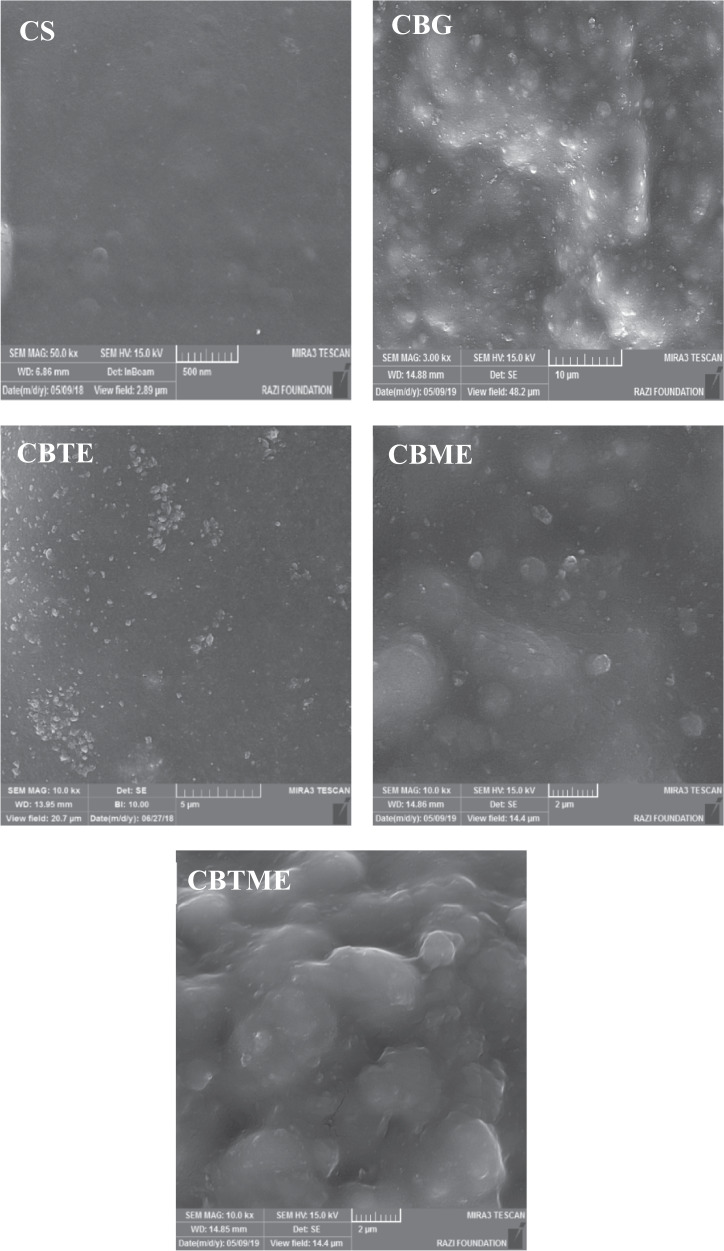
FE-SEM images of CS, CBG, CBTE, CBME and CBTME coatings (SEM magnification in all images is 10.0 kx)

Figure 1A shows FE-SEM images of the biomedical grade Titanium sheets after hydrothermal treatment. After hydrothermal treatment, sphere shape structures were then observed. Surface roughness of the sample after hydrothermally etching was increased (Fig. 1B). Grazing incidence X-ray diffraction (GIXRD) pattern of surface after etching was shown in Fig. 1C. The reflection patterns of substrate after etching was matched with JCPDS card number 88-1175 corresponding to rutile structure. The preferable direction of TiO_2_ formation phase is on (200) plane 2Teta: 39.8. The preferable growth direction was determined by the most dominate reflections of the crystal planes. The morphology of coatings deposited on hydrothermally etched substrate is shown in Fig. 2.

**Fig. 3:**
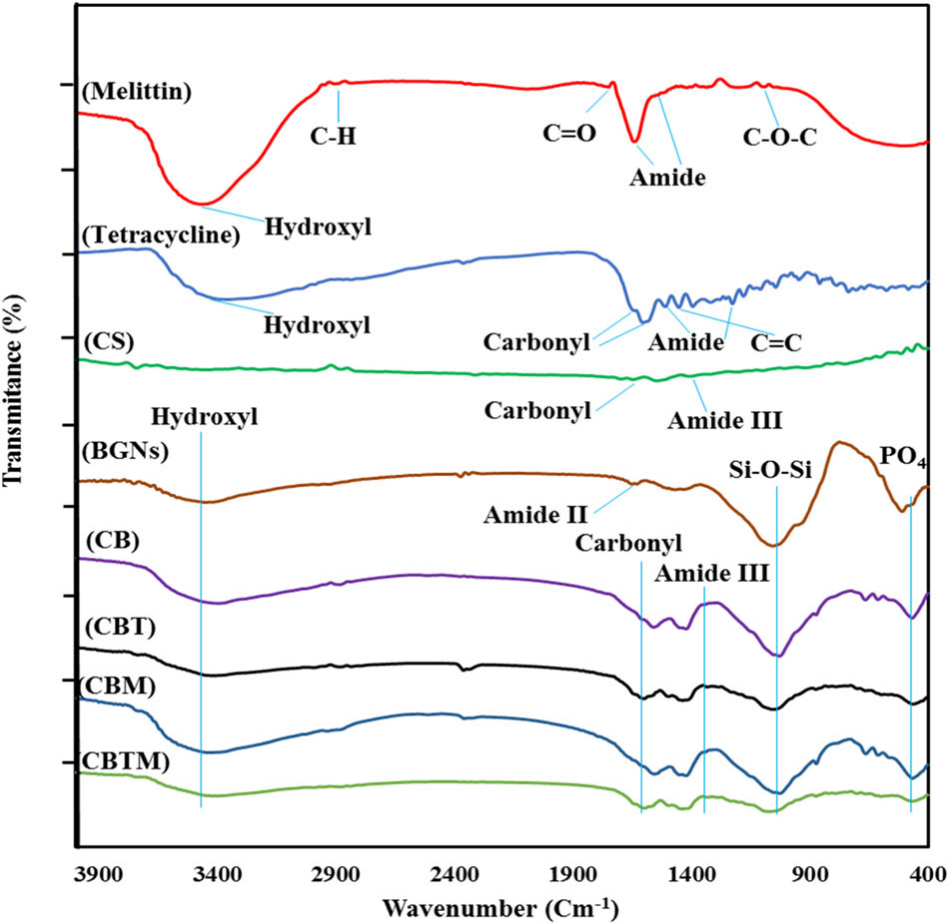
FTIR spectra of pristine components and composite coatings

Figure 3 shows the FTIR spectra of CS, CB, CBT, CBM and CBTM coatings and those components. The functional groups of tetracycline, bioactive glass and chitosan were revealed in composite coatings but Melittin functional groups didn’t detect in composite coatings. Melittin amount was very low, thus its functional groups didn’t reveal in FTIR spectra of CBM and CBTM coatings. The broad bonds at 3450 cm^−1^, 2900 cm^−1^, 1659 cm^−1^ and 1320 cm^−1^ are attributed to Hydroxyl group, C–H stretching, carbonyl group and amide III bond, respectively [48, 49]. The absorption bonds at 1055 and 877 cm^−1^ are attributed to Si-O-Si group in composite coatings [50]. Also the absorption bond at around 467 cm^−1^ is corresponding to PO_4_ [38]. Slight changes in the peak position of bioactive glass, chitosan and tetracycline were noticed in CB and CBT and CBTM composite coatings that could be attributed to hydrogen bonds formation between hydroxyl and amine groups of CS and tetracycline and oxygen of the glass network [51].

### Proliferation and differentiation of MC3T3-E1 cells

**Fig. 4:**
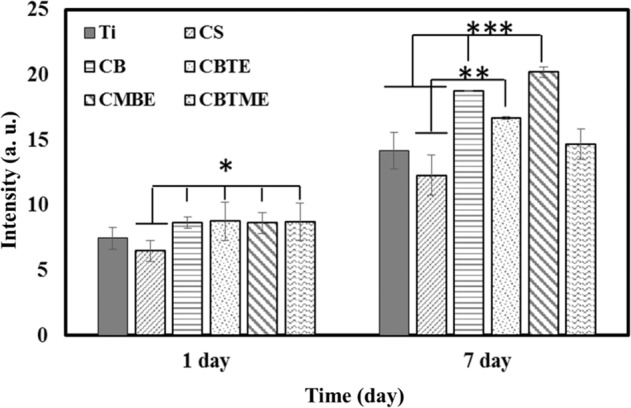
Alamar blue assay of experimental groups after 1 and 7 days (*P < 0.05, **P < 0.01, ***P < 0.001)

**Fig. 5:**
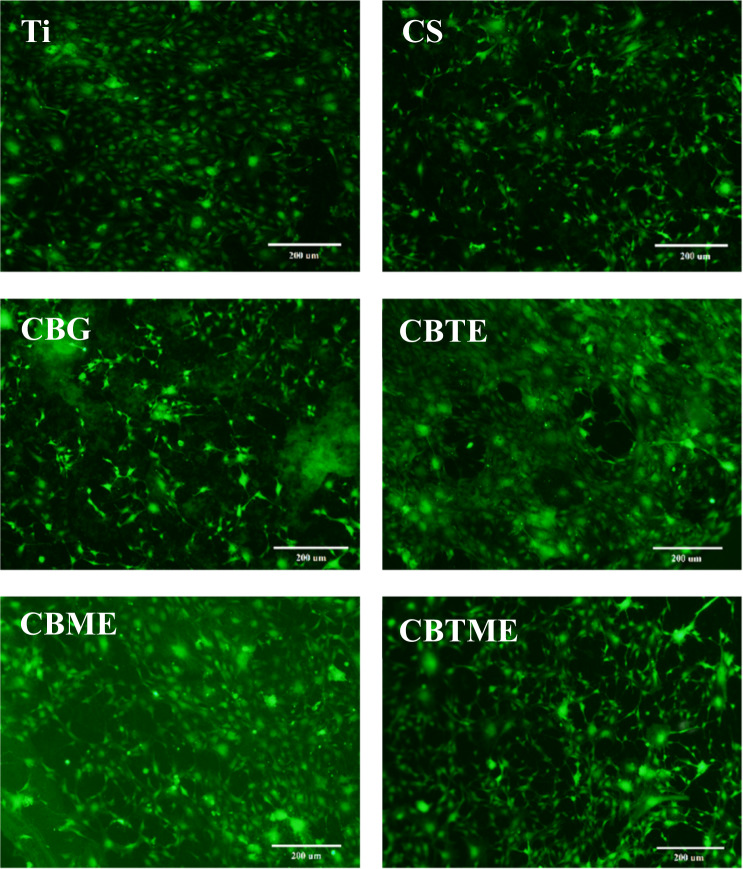
Fluorescence images after live/dead staining of MC3T3 cells cultured for 2 days on experimental groups

The proliferation of MC3T3 cells on experimental groups was assayed by Alamar Blue test. After the first day of incubation, no significant differences in cell proliferation were observed compared to the substrate (Fig. 4). In the CS group, the cell proliferation was lower than Ti group however the difference was not significant, while the difference of cell growth in the CS group in comparison with composite coatings was significant (Fig. 4). The cells that were cultured on composite coatings proliferated more rapidly than those cultured on CS and Ti groups, after 7 days. Also, bioactive glass in composite coatings accelerated cell proliferation. Melittin and tetracycline in composite coatings have positive effect on cell proliferation compared to CS and Ti groups. Fluorescence images after live/dead staining of MC3T3 cells cultured for 2 days on experimental groups were shown in Fig. 5. The density of live MC3T3 cells on all experimental groups except for the CS coating was similar.

**Fig. 6:**
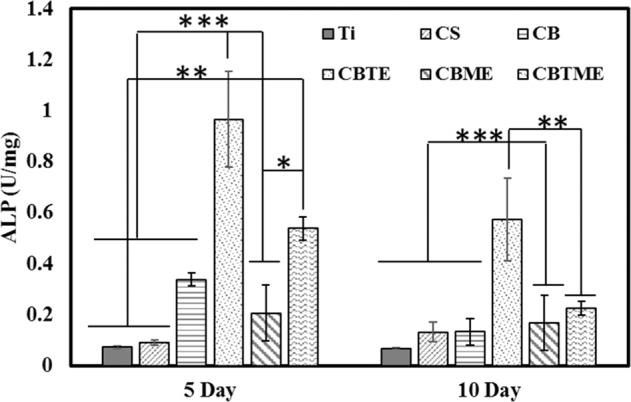
Normalized ALP assays of MC3T3 cells cultured on the surfaces after 5 and 10 days (*P < 0.05, **P < 0.01, ***P < 0.001)

The secretion of ALP by MC3T3 cells that were cultured on CBTE coating was significantly higher than other groups after 5 and 10 days (Fig. 6). ALP secretion in CBG, CBME and CBTME coatings was higher than CS and Ti groups. ALP levels did not increase significantly in Ti and CS groups during 10 days (Fig. 6). These results demonstrated that composite coating causes MC3T3 to differentiate into osteoblasts and this differentiation increases when bioactive glass, tetracycline and Melittin are used as one of coating’s component.

### Antibacterial performance

**Fig. 7:**
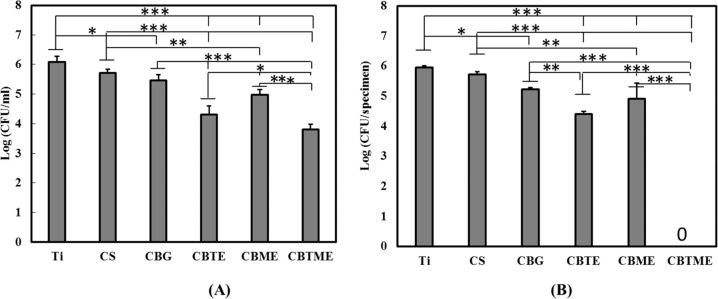
**A** The numbers of planktonic bacteria. **B** The numbers of adhered bacteria on different surfaces (*P < 0.05, **P < 0.01, ***P < 0.001)

**Fig. 8:**
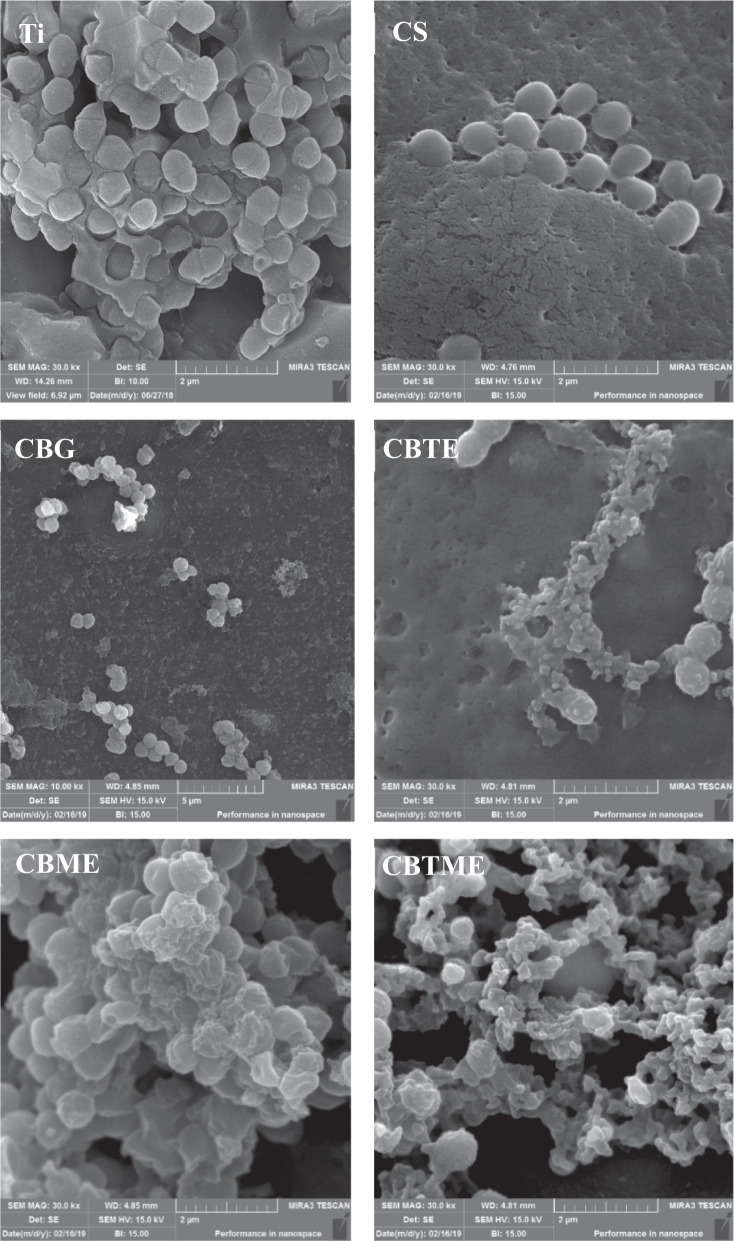
SEM images of MRSA bacteria on experimental groups after incubation for 6 h (SEM magnification in all images is 30.0 kx)

Antibacterial activities of the experimental groups were estimated after 6 h for both planktonic and adherent MRSA bacteria (Fig. 7A, B). CBTME coating eradicated adherent bacteria because of the synergistic effect of tetracycline and Melittin. Also, the numbers of planktonic bacteria in CBTME decreased over 3 logs compared to Ti. Reduction of both planktonic and adherent bacteria was seen in CS, CBTE and CBME coatings. SEM images of MRSA bacteria on the surfaces were shown in Fig. 8. On the CBTE, CBME and CBTME coatings, destroyed and live bacteria were seen. More bacteria were killed on CBTME coating group. Ti, CS and CB coatings were exhibited normal morphology of live MRSA bacteria without any destruction.

## Discussion

### The mechanism of coating deposition

Chitosan is a linear copolymer of glucosamine and N-acetylglucosamine that in acidic solutions, behaves as a cationic polyelectrolyte. By evaporating water from acidic solutions of chitosan, strong chitosan/ bioactive glass nanoparticles composite uniform coatings are easily formed due to the presence of hydrogen bonds between the hydroxyl and amino groups of chitosan and TiO2 layer on the etched surface and the oxygen of the bioactive glass network. FTIR results showed that tetracycline moleculs didn’t have strong bond with chitosan matrix and were released by chitosan degradation or swelling [52].

### Bone regeneration capacity

In the present study we used bioactive glass nanoparticles as osteogenic agent. So, it increased ALP enzyme of MC3T3 cells and showed differentiation of them to osteoblast cells. But, the attractive result of the present research is synergism effect of tetracycline with bioactive glass on ALP secretion. Tetracycline antibiotic with chemotactic effect and anti-collagenolytic activity has guided bone regenerating potential [35]. Tetracycline with anti-inflammatory and inhibition of osteoclast function guides bone regeneration [35, 36]. Therefore, it is widely used in periodontal therapy; because of its multifunctional behavior include bone regeneration and antibacterial activity [36, 53]. Increasing ALP enzyme of MC3T3 cells by tetracycline revealed that it is a suitable candidate as osteoinductive and antibacterial agent in orthopedic implants coatings. Although, Melittin increased the proliferation of MC3T3 cells but didn’t have positive synergism on ALP production in CBME and CBTME coatings.

### Antibacterial performance

The “race for the surface,” phrase points to tissue cell integration and bacterial adhesion compete for colonization on the implant’s surface [54]. Having antiadhesive or bactericidal surface for implants is necessary. Synergism of Melittin and tetracycline made antiadhesive or bactericidal surface that can eradicate adherent bacteria. So, formation of biofilm was prevented in decisive period in early stages and colonization of bone cells occurred. Killing mechanism of Melittin was destruction of bacterial membrane [31], while, tetracycline binds to bacterial ribosomes and prevents the attachment of the aminoacyl tRNA to the RNA-ribosome complex. It simultaneously inhibits other steps of the protein biosynthesis [55]. It is supposed that the penetration of tetracycline is facilitated by increasing the membrane permeability after Melittin exposure.

Chitosan and bioactive glass have antibacterial properties that influence on bactericidal properties of coatings. Amino groups of chitosan disrupt the bacterial membrane and killed them [56, 57]. Release of alkaline ions from bioactive glass increases the pH of medium. Bacteria can’t tolerate higher pH and die [58].

### Clinical perspective

Antibiotic resistance is rising to dangerously high levels in all parts of the world. Antibiotic resistance leads to higher medical costs, prolonged hospital stays, and increased morbidity and mortality. The world urgently needs to change the way it prescribes and uses antibiotics. The suggestion of present study is pretreatment of orthopedic implants by combination of tetracycline and Melittin.

## Conclusions

In the present study, titanium substrate was etched hydrothermally in H_2_O_2_ solution. Then, chitosan/bioactive glass nanoparticles/ tetracycline composite coatings were coated on etched substrate. After composite coating, Melittin as an antimicrobial peptide was drop-casted on the surface of specimens. The surfaces were assessed in vitro using both biocompatibility and antimicrobial assays. As a conclusion, tetracycline has antibacterial and osteogenic properties simultaneously. The combination of tetracycline and Melittin in the coatings decreased both planktonic and adherent MRSA bacteria, significantly. Combination of tetracycline and Melittin in the coatings eradicated adherent bacteria and prevented biofilm formation on the surface of implant.
